# Supplementary material for: Complete Mitochondrial Genome of Scolytoplatypodini Species (Coleoptera: Curculionidae: Scolytinae) and Phylogenetic Implications
Source: Genes (Basel). 2023 Jan 6;14(1):162. doi: 10.3390/genes14010162 (PMC9859420; doi:10.3390/genes14010162)
Supplement: Supplementary file 1 [file genes-14-00162-s001.zip › Supplementary Tables and Figures.pdf]

Supplementary Data:
Table S1. The best-fit partitioning scheme and corresponding models used on the phylogenetic tree.

Partition names	Sites	Best Model
charset Subset1 = 2344-3021\3, 1-666\3, 3022-3801\3, 3802-4932\3 (*cox2* codon1, *atp6* codon1, *cox3* codon1, *cytb* codon1)	1085	GTR+I+G
charset Subset2 = 818-2343\3, 2345-3021\3, 3023-3801\3, 3803-4932\3, 2-666\3 (*cox1* codon2, *cox2* codon2, *cox3* codon2, *cytb* codon2, *atp6* codon2)	1594	TVM+I+G
charset Subset3 = 6828-7170\3, 3-666\3, 819-2343\3, 3804-4932\3, 669-816\3, 2346-3021\3, 3024-3801\3 (*nad3* codon3, *atp6* codon3, *cox1* codon3, *cytb* codon3, *atp8* codon3, *cox2* codon3, *cox3* codon3)	1759	GTR+G
charset Subset4 = 6826-7170\3, 5869-6825\3, 10405-10875\3, 668-816\3, 667-816\3 (*nad3* codon1, *nad2* codon1, *nad6* codon1, *atp8* codon2, *atp8* codon1)	691	GTR+I+G
charset Subset5 = 817-2343\3 (*cox1* codon1)	509	GTR+I+G
charset Subset6 = 4933-5868\3, 7171-7449\3, 8758-10404\3, 7450-8757\3 (*nad1*codon1, *nad4L* codon1, *nad5* codon1, *nad4* codon1)	1390	GTR+I+G
charset Subset7 = 7451-8757\3, 4934-5868\3, 7172-7449\3, 8759-10404\3 (*nad4* codon2, *nad1* codon2, *nad4L* codon2, *nad5* codon2)	1390	GTR+I+G
charset Subset8 = 7452-8757\3, 8760-10404\3, 4935-5868\3, 7173-7449\3 (*nad4* codon3, *nad5* codon3, *nad1* codon3, *nad4L* codon3)	1390	TRN+G
charset Subset9 = 10406-10875\3, 5870-6825\3, 6827-7170\3 (*nad6* codon2, *nad2* codon2, *nad3* codon2)	591	TVM+I+G
charset Subset10 = 10407-10875\3, 5871-6825\3 (*nad6* codon3, *nad2* codon3)	476	TRN+G

Table S2. Nucleotide compositions in regions of the *S. raja*, *S. sinensis*, *S. skyliuae,* and *S. wugongshanensis* mitochondrial genomes.

Species	Gene Region	Size (bp)	A(%)	T(%)	C(%)	G(%)	A+T(%)	G+C(%)	AT-Skew	GC-Skew
S. raja	Whole mitogenome	15324	39.4	36.4	14.8	9.4	75.8	24.2	0.04	-0.22
	13PCGs	11070	31.9	41.8	13.7	12.6	73.7	26.3	-0.13	-0.04
	1st coden	3690	34.5	33.6	14.2	17.7	68.1	31.9	0.01	0.11
	2st coden	3690	20.8	48.4	17.1	13.7	69.2	30.8	-0.40	-0.11
	3st coden	3690	40.3	43.5	9.9	6.3	83.8	16.2	-0.04	-0.22
	tRNA genes	1437	40.8	39.5	8.1	11.6	80.3	19.7	0.02	0.18
	rRNA genes	2065	38.9	42.6	5.9	12.6	81.5	18.5	-0.05	0.36
	D-loop	586	43.5	37.7	13.3	5.5	81.2	18.8	0.07	-0.41
S. sinensis	Whole mitogenome	15394	39.5	36.3	15.0	9.2	75.8	24.2	0.04	-0.24
	13PCGs	11062	31.6	42.0	13.7	12.7	73.6	26.4	-0.14	-0.04
	1st coden	3688	34.3	34.2	13.8	17.7	68.5	31.5	0.00	0.12
	2st coden	3687	20.8	48.3	17.1	13.8	69.1	30.9	-0.40	-0.11
	3st coden	3687	39.7	43.5	10.2	6.6	83.2	16.8	-0.05	-0.21
	tRNA genes	1445	41.1	39.0	8.6	11.3	80.1	19.9	0.03	0.14
	rRNA genes	2073	38.3	42.6	6.1	13.0	80.9	19.1	-0.05	0.36
	D-loop	621	47.0	38.3	11.5	3.2	85.3	14.7	0.10	-0.56
S. skyliuae	Whole mitogenome	15167	39.6	36.7	15.5	8.2	76.3	23.7	0.04	-0.31
	13PCGs	11023	32.3	42.2	13.6	11.9	74.5	25.5	-0.13	-0.07
	1st coden	3675	35.8	34.3	13.5	16.4	70.1	29.9	0.02	0.10
	2st coden	3674	20.6	48.3	17.3	13.8	68.9	31.1	-0.40	-0.11
	3st coden	3674	40.5	44.1	9.8	5.6	84.6	15.4	-0.04	-0.27
	tRNA genes	1424	41.7	39.7	8.2	10.4	81.4	18.6	0.02	0.12
	rRNA genes	2056	38.6	41.7	5.5	14.2	80.3	19.7	-0.04	0.44
	D-loop	495	44.4	37.4	15.4	2.8	81.8	18.2	0.09	-0.69
S. wugongshanensis	Whole mitogenome	15267	39.0	37.2	15.6	8.2	76.2	23.8	0.02	-0.31
	13PCGs	11025	32.0	42.3	13.7	12.0	74.3	25.7	-0.14	-0.07
	1st coden	3675	35.1	35.3	12.8	16.8	70.4	29.6	0.00	0.14
	2st coden	3675	20.8	48.9	16.7	13.6	69.7	30.3	-0.40	-0.10
	3st coden	3675	40.1	42.9	11.3	5.7	83.0	17.0	-0.03	-0.33
	tRNA genes	1413	41.8	39.1	8.5	10.6	80.9	19.1	0.03	0.11
	rRNA genes	2044	39.4	41.1	5.5	14.0	80.5	19.5	-0.02	0.44
	D-loop	670	42.5	40.6	13.8	3.1	83.1	16.9	0.02	-0.63

Table S3. Mitogenomic characteristics of *S. raja*/*S. sinensis*/*S. skyliuae*/*S. wugongshanensis*.

Gene	Strand	Position		Codons		Anticodon	Size (bp)	IGN
		From	To	Start	Stop			
trnI	J	1/1/1/1	67/67/66/65			ACT	67/67/66/65	0/0/0
trnQ	N	68/64/94/77	138/137/161/144			CAA	71/71/68/68	0/4/27/11
trnM	J	137/133/161/145	204/200/228/211			ATG	68/68/68/67	-2/2/-1/0
nad2	J	205/201/259/212	1212/1208/1245/1228	ATT/ATT/ATA/ATC	TAA/TAA/TAA/TAA		1008/1008/987/1017	0/0/30/0
trnW	J	1211/1207/1246/1228	1279/1275/1311/1292			TGA	68/69/66/65	-2/-2/0/-1
trnC	N	1279/1275/1313/1292	1341/1338/1377/1358			TGC	63/64/65/67	-1/-1/1/-1
trnY	N	1342/1339/1378/1359	1400/1397/1437/1417			TAC	59/59/60/59	0/0/0/0
cox1	J	1411/1413/1440/1453	2949/2951/2978/2955	ATT/ATT/ATT/ATT	TAA/TAA/TAA/TAA		1539/1539/1539/1503	10/15/2/35
trnL2	J	2956/2963/2992/2963	3019/3026/3054/3025			TTA	64/64/63/63	6/11/13/7
cox2	J	3020/3027/3055/3026	3703/3710/3736/3709	ATC/ATT/ATT/ATT	TAA/TAA/T--/TAA		684/684/682/684	0/0/0/0
trnK	J	3717/3723/3737/3713	3785/3791/3804/3782			AAG	69/69/68/70	13/12/0/3
trnD	J	3785/3792/3805/3782	3847/3854/3867/3844			GAC	63/63/63/63	1/0/0/-1
atp8	J	3848/3855/3876/3845	4009/4016/4028/4003	ATC/ATC/ATA/ATC	TAA/TAA/TAA/TAA		162/162/153/159	0/0/8/0
atp6	J	4003/4010/4022/3997	4669/4676/4688/4663	GTG/ATG/ATG/ATG	T--/T--/T--/T--		667/667/667/667	-7/-7/-7/-7
cox3	J	4670/4677/4689/4664	5449/5456/5468/5443	ATG/ATG/ATG/ATG	TAA/TAG/TAA/TAA		780/780/780/780	0/0/0/0
trnG	J	5473/5507/5477/5449	5535/5569/5540/5512			GGA	63/63/64/64	23/50/8/5
nad3	J	5542/5576/5547/5518	5889/5923/5894/5865	ATT/ATT/ATT/ATT	TAA/TAA/TAA/TAA		348/348/348/348	6/6/6/5
trnA	J	5898/5939/5895/5865	5961/6003/5958/5926			GCA	64/65/64/62	8/15/0/-1
trnN	J	5962/6002/5958/5926	6026/6066/6024/5990			AAC	65/65/67/65	0/-2/-1/-1
trnR	J	6041/6073/6033/6001	6104/6137/6097/6064			CGA	64/65/65/64	14/6/8/10
trnS1	J	6105/6138/6099/6065	6163/6197/6153/6121			AGA	59/60/55/57	0/0/1/0
trnE	J	6164/6198/6156/6123	6225/6260/6219/6186			GAA	62/63/64/64	0/0/2/1
trnF	N	6225/6260/6218/6185	6289/6324/6281/6249			TTC	65/65/64/65	-1/-1/-2/-2
nad5	N	6291/6334/6283/6250	8000/8043/7989/7962	ATT/ATT/ATATATA	TAA/TAG/TAA/TAA		1710/1710/1707/1713	1/9/1/0
trnH	N	8001/8044/7990/7963	8064/8108/8055/8026			CAC	65/65/66/64	0/0/0/0

nad4	N	8069/8112/8055/8026	9388/9431/9374/9345	ATG/ATG/ATG/ATG	TAG/TAG/TAA/TAA		1320/1320/1320/1320	4/3/-1/-1
nad4l	N	9382/9425/9368/9339	9672/9751/9658/9629	ATG/ATG/ATG/ATG	TAA/TAA/TAA/TAA		291/291/291/291	-7/-7/-7/-7
trnT	J	9678/9721/9677/9632	9742/9787/9740/9694			ACA	65/67/64/63	5/5/18/2
trnP	N	9743/9788/9741/9695	9810/9855/9805/9757			CCA	68/68/65/63	0/0/0/0
nad6	J	9819/9864/9813/9765	10322/10367/10313/10262	ATT/ATA/ATT/ATT	TAA/TAA/TAA/TAA		504/504/501/498	8/8/7/7
cob	J	10316/10367/10310/10259	11437/11479/11425/11371	ATC/ATG/ATA/ATA	TAA/TAA/TAA/TAA		1122/1113/1116/1113	-7/-1/-4/-4
trnS2	J	11465/11505/10430/11376	11532/11573/11496/11441			TCA	68/69/67/66	27/25/4/4
nad1	N	11601/11629/11550/11490	12536/12564/12482/12422	TTG/TTG/ATA/ATG	TAA/TAA/TAA/TAA		936/936/933/933	68/55/53/48
trnL1	N	12537/12565/12484/12425	12603/12630/12550/12489			CTA	67/66/67/65	0/0/1/2
rrnL	N	12604/12631/12551/12490	13915/13943/13850/13779				1312/1313/1300/1290	0/0/0/0
trnV	N	13916/13944/13851/13780	13985/14013/13915/13843			GTA	70/70/65/64	0/0/0/0
rrnS	N	13986/14014/13916/13844	14738/14773/14671/14597				753/760/756/754	0/0/0/0
D-loop		14739/14774/14672/14598	15324/15394/15166/15267				586/621/495/670	0/0/0/0

NOTE: N, N-strand; J, J-strand; IGN = intergenic nucleotides; – not determined.

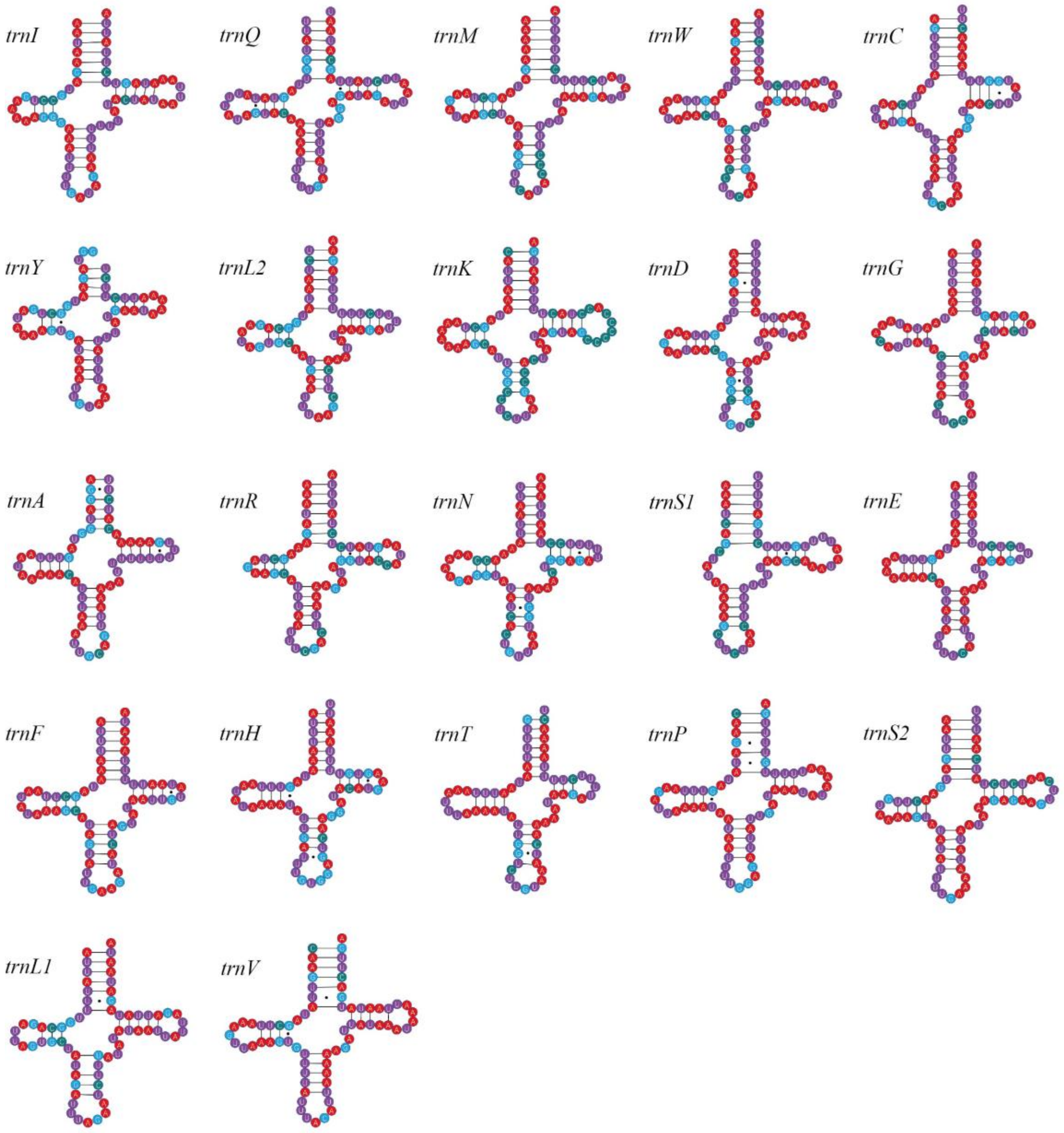

Figure S1. Secondary structures for tRNA genes from the mtDNA of *S. raja*.

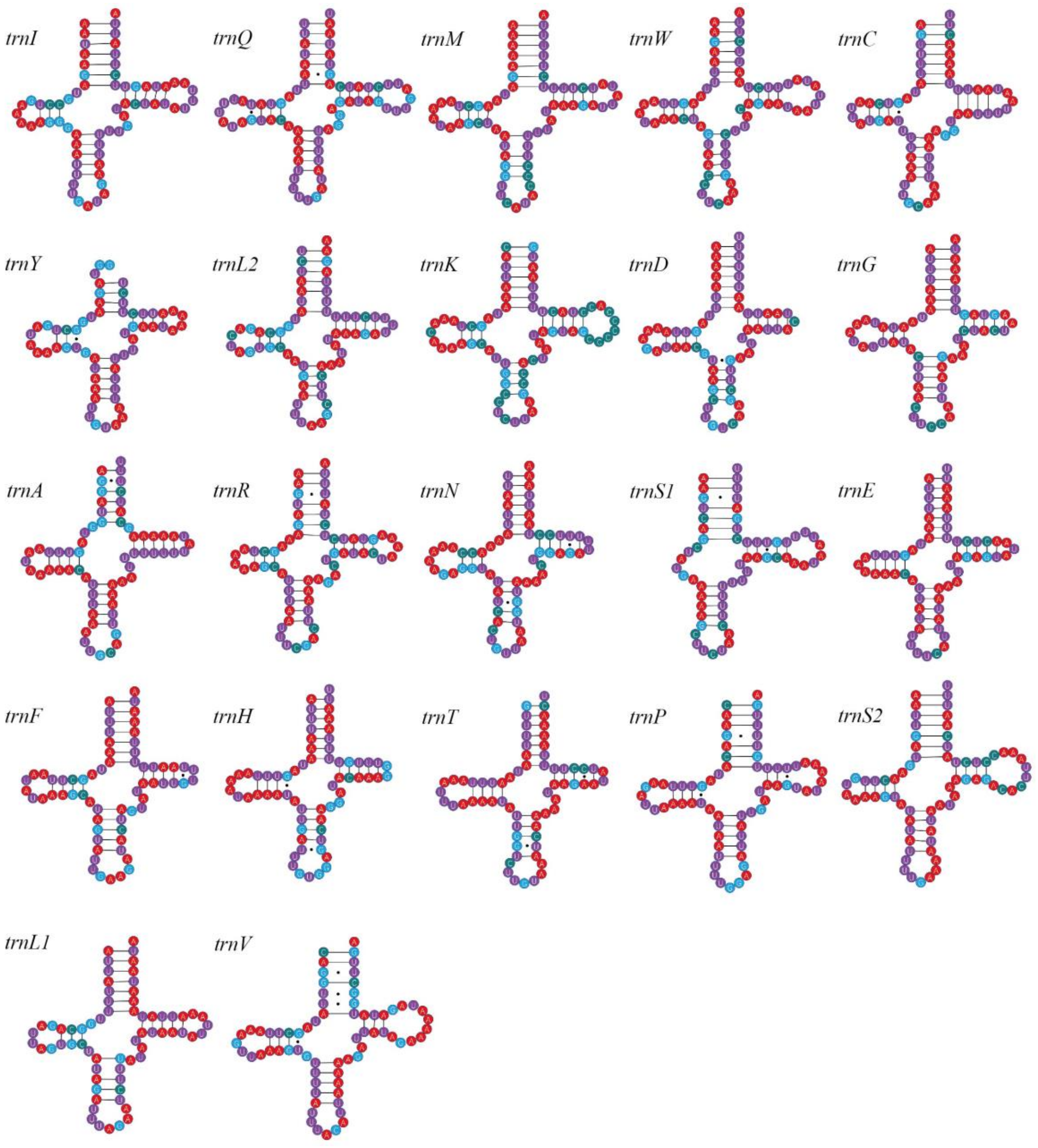

Figure S2. Secondary structures for tRNA genes from the mtDNA of *S. sinensis*.

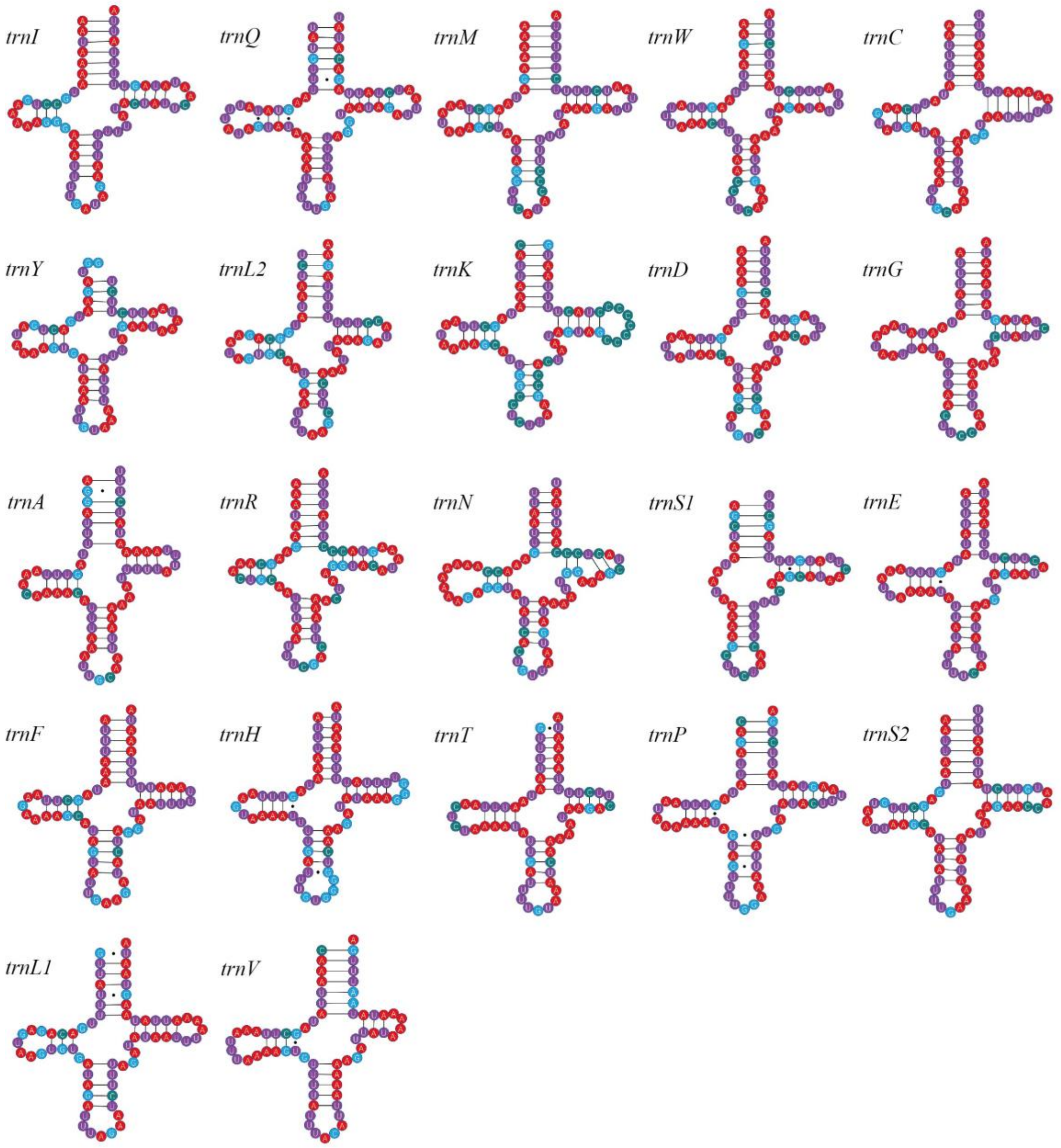

Figure S3. Secondary structures for tRNA genes from the mtDNA of *S. skyliuae*.

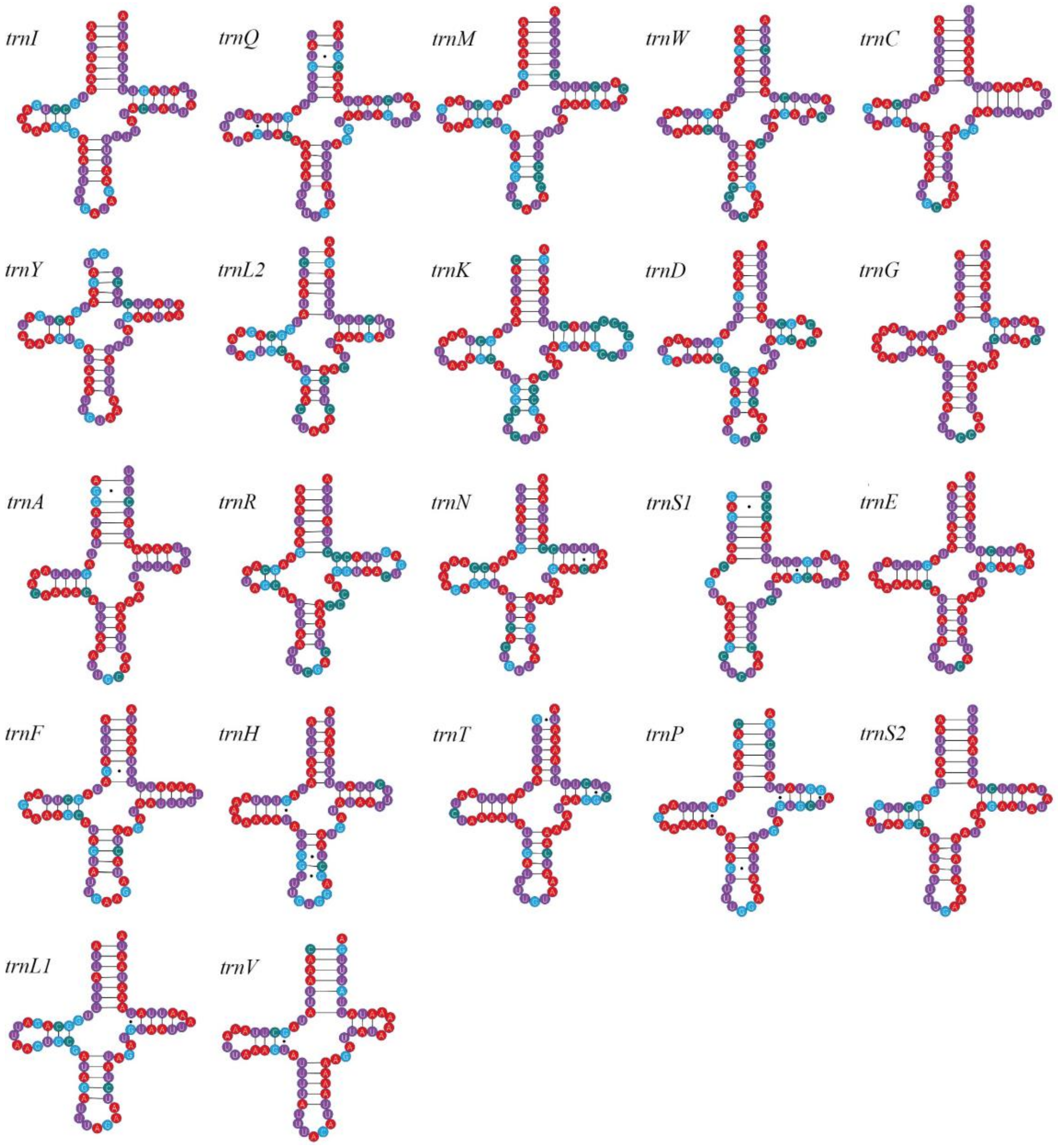

Figure S4. Secondary structures for tRNA genes from the mtDNA of *S. wugongshanensis*.

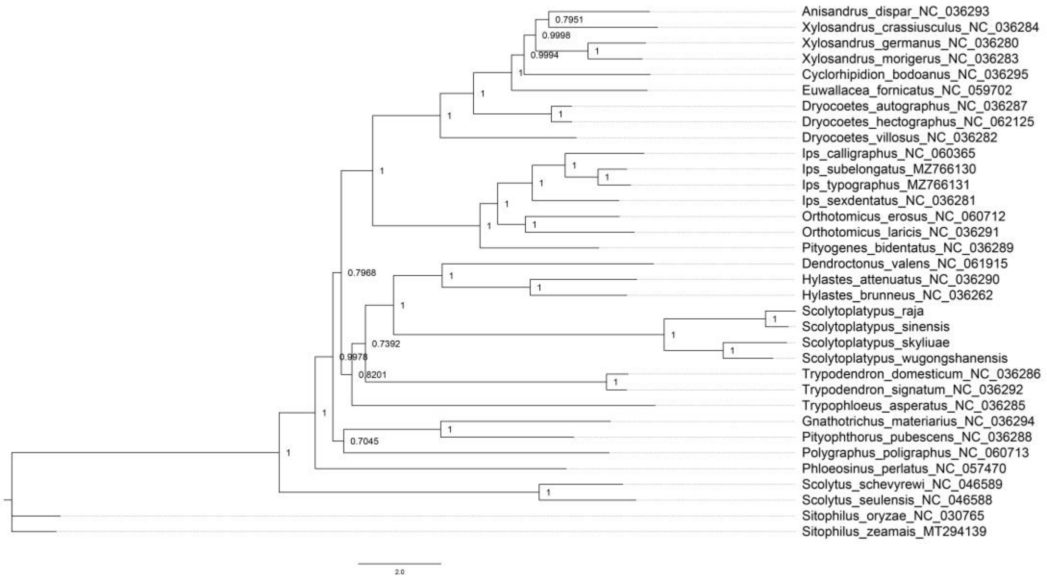

Figure S5. Phylogenetic tree of Scolytinae using the BI method based on 13 PCGs.
